# Supplementary material for: Nitrogen-fixing trees could exacerbate climate change under elevated nitrogen deposition
Source: Nat Commun. 2019 Apr 2;10:1493. doi: 10.1038/s41467-019-09424-2 (PMC6445091; doi:10.1038/s41467-019-09424-2)
Supplement: Supplementary file 1 — Supplementary Information [file 41467_2019_9424_MOESM1_ESM.pdf]

Nitrogen-fixing trees could exacerbate climate change under elevated nitrogen deposition

Supplementary Information

Kou-Giesbrecht and Menge

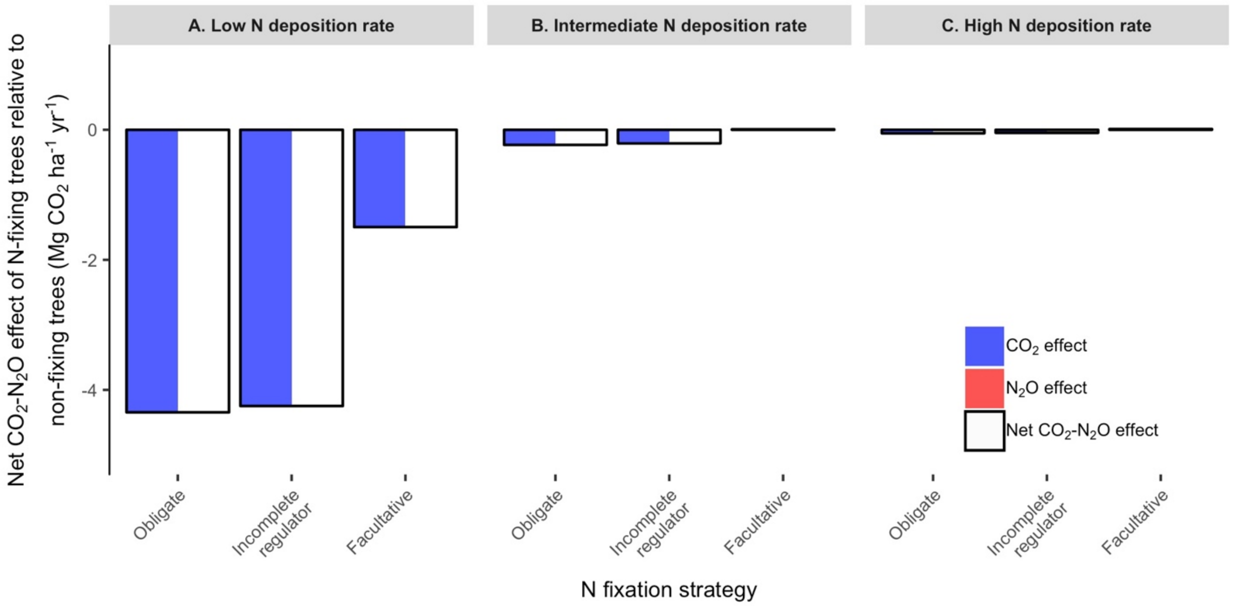

Supplementary Figure 1: CO_2 and N_2O effects of nitrogen-fixing trees relative to non-fixing trees. a. The CO_2 and N_2O effects of N-fixing trees relative to non-fixing trees under low N deposition rates. b. The CO_2 and N_2O effects of N-fixing trees relative to non-fixing trees under intermediate N deposition rates. c. The CO_2 and N_2O effects of N-fixing trees relative to non-fixing trees under high N deposition rates. The details of this figure are the same as those for Figure 1, except that the model had a temperate forest parameterization instead of a tropical forest parameterization.

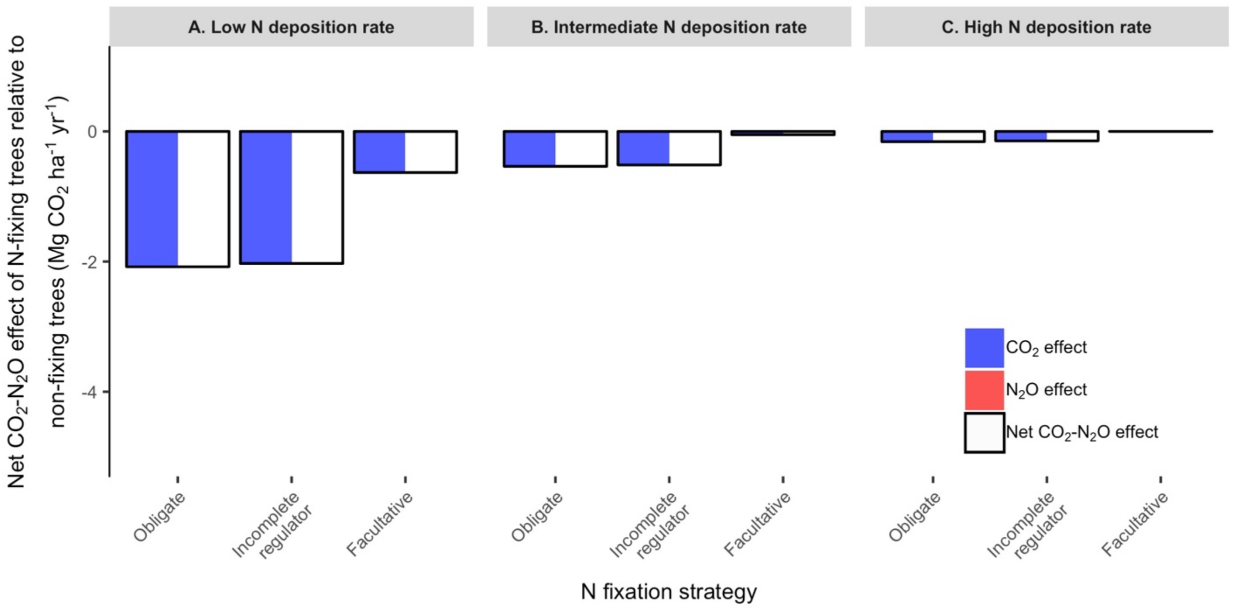

Supplementary Figure 2: CO_2 and N_2O effects of nitrogen-fixing trees relative to non-fixing trees. a. The CO_2 and N_2O effects of N-fixing trees relative to non-fixing trees under low N deposition rates. b. The CO_2 and N_2O effects of N-fixing trees relative to non-fixing trees under intermediate N deposition rates. c. The CO_2 and N_2O effects of N-fixing trees relative to non-fixing trees under high N deposition rates. The details of this figure are the same as those for Figure 1, except that the model had a boreal forest parameterization instead of a tropical forest parameterization.

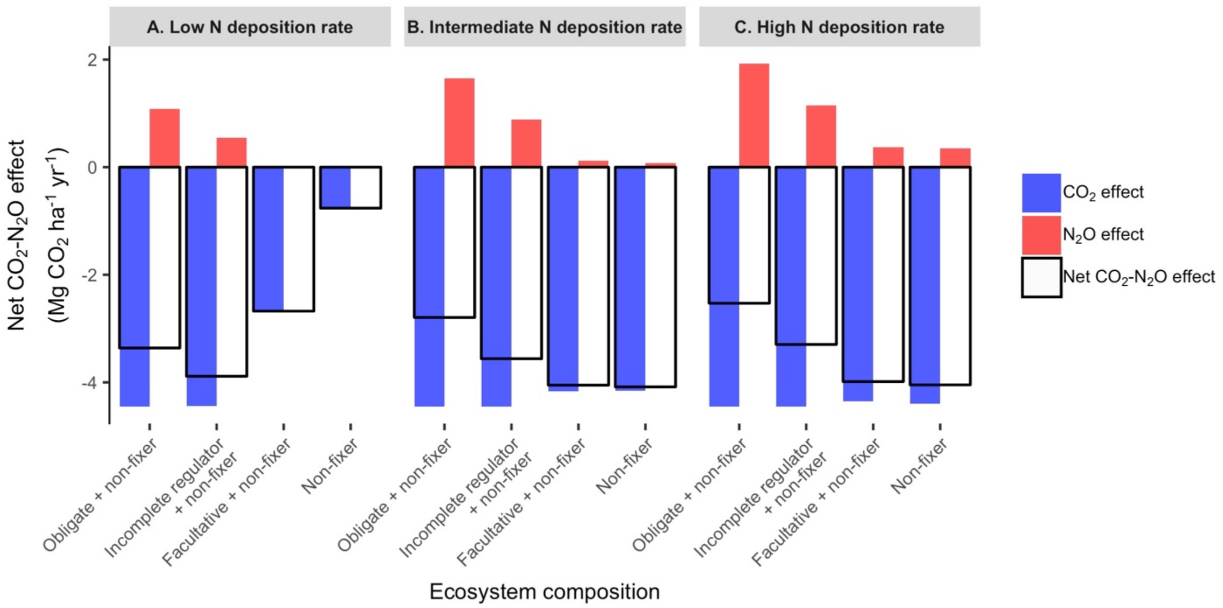

Supplementary Figure 3: CO_2 and N_2O effects of ecosystems. a. The CO_2 and N_2O effects of ecosystems under low N deposition rates. b. The CO_2 and N_2O effects of ecosystems under intermediate N deposition rates. c. The CO_2 and N_2O effects of ecosystems under high N deposition rates. The details of this figure are similar to those for Figure 1, except that this figure shows the net CO_2-N_2O effect of each ecosystem, whereas Figure 1 shows the difference in net CO_2-N_2O effects between ecosystems that contain both N-fixing trees and non-fixing trees (three left-most bars within each N deposition panel) and ecosystems of non-fixing trees (right-most bar within each N deposition panel).

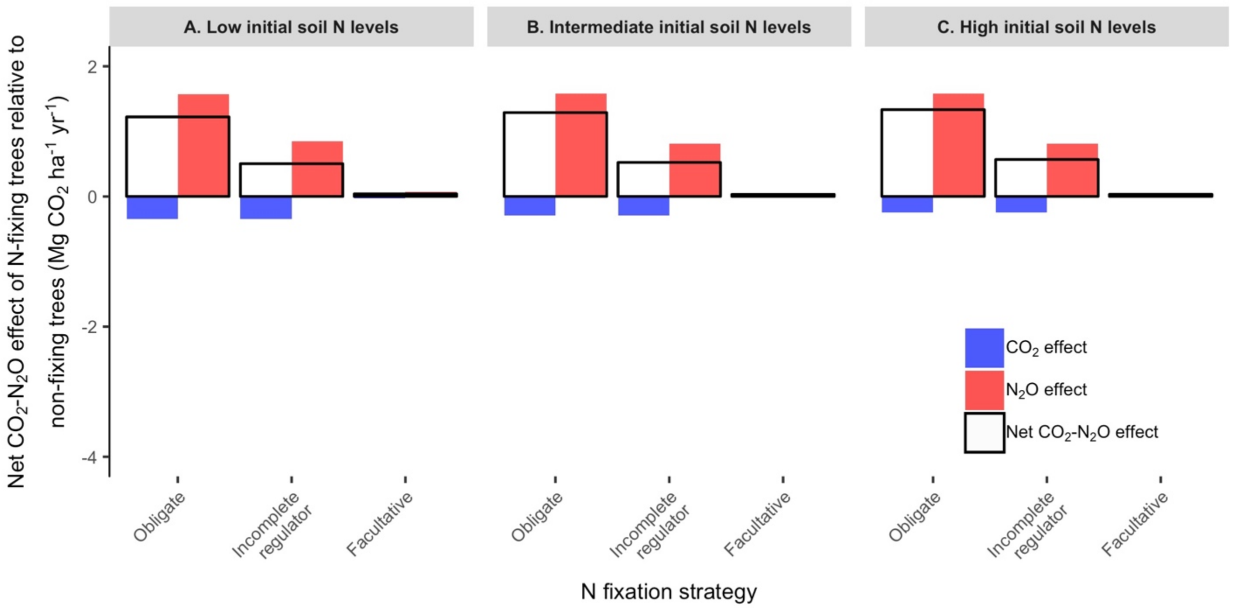

Supplementary Figure 4: CO_2 and N_2O effects of nitrogen-fixing trees relative to non-fixing trees. a. The CO_2 and N_2O effects of N-fixing trees relative to non-fixing trees for low initial soil N levels. b. The CO_2 and N_2O effects of N-fixing trees relative to non-fixing trees for intermediate soil N levels. c. The CO_2 and N_2O effects of N-fixing trees relative to non-fixing trees for high soil N levels. The details of this figure are similar to those for Figure 1, except that this figure shows the net CO_2-N_2O effects of N-fixing trees relative to non-fixing trees for different initial soil N levels (for an intermediate N deposition rate[1]), whereas Figure 1 shows the net CO_2-N_2O effects of N-fixing trees relative to non-fixing trees for different N deposition rates (for an intermediate initial soil N level). See Supplementary Table 4 for the parameterization of initial soil N levels.

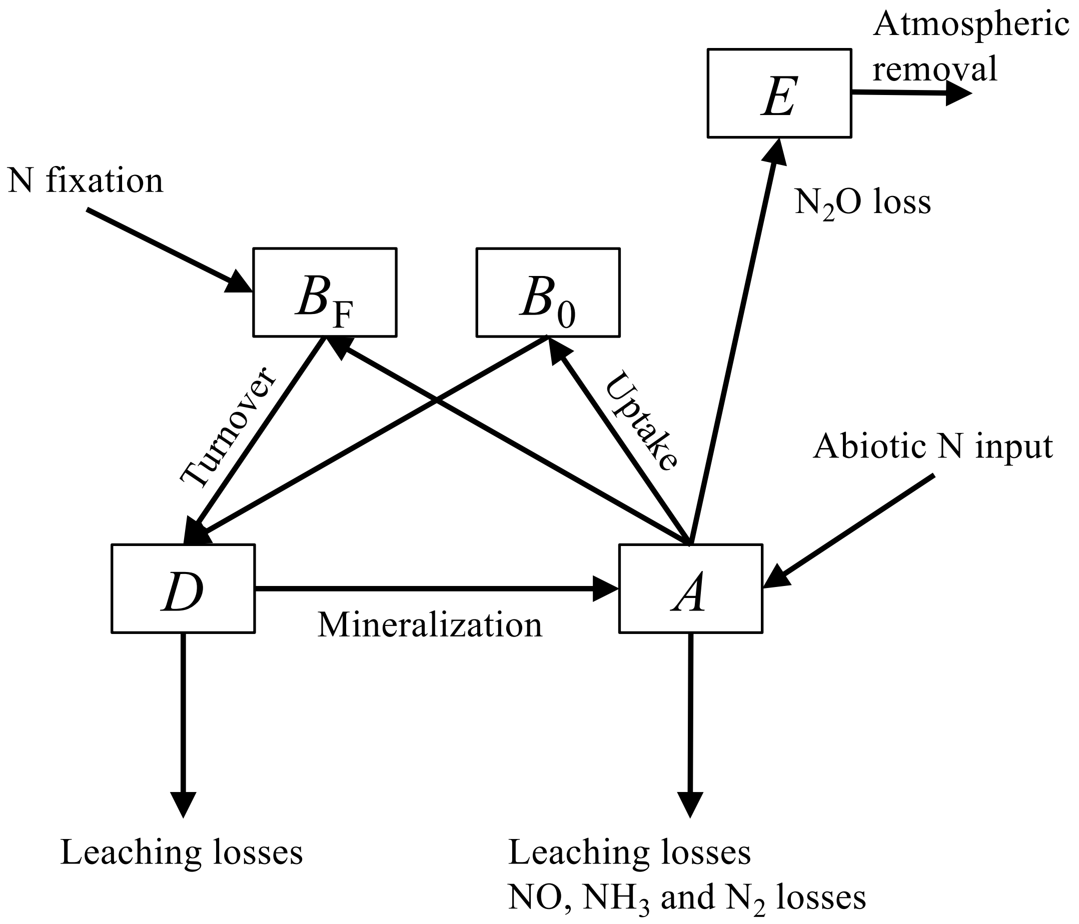

Supplementary Figure 5: Box diagram of model. Boxes represent nutrient pools in the ecosystem, and arrows represent nutrient fluxes in the ecosystem. B_F is the N-fixer biomass C pool, B_0 is the non-fixer biomass C pool, *D* is the plant-unavailable soil N pool, *A* is the plant-available soil N pool, and *E* is the atmospheric N_2O pool.

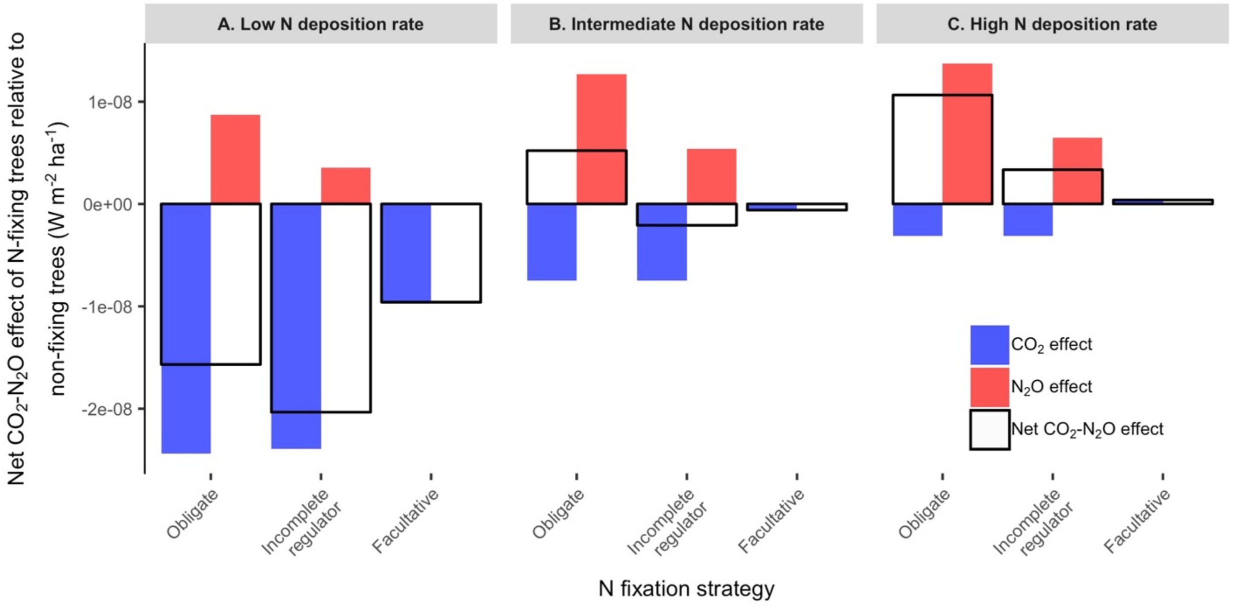

Supplementary Figure 6: CO_2 and N_2O effects of nitrogen-fixing trees relative to non-fixing trees. a. The CO_2 and N_2O effects of N-fixing trees relative to non-fixing trees under low N deposition rates. b. The CO_2 and N_2O effects of N-fixing trees relative to non-fixing trees under intermediate N deposition rates. c. The CO_2 and N_2O effects of N-fixing trees relative to non-fixing trees under high N deposition rates. This figure differs from Figure 1 in that it shows net CO_2-N_2O effects of N-fixing trees relative to non-fixing trees as the net radiative forcing from continuous CO_2 sequestration and soil N_2O emissions over 100 years of ecosystem succession, whereas Figure 1 shows the net CO_2-N_2O effects of N-fixing trees relative to non-fixing trees as accumulated CO_2 sequestration compared to accumulated soil N_2O emissions after 100 years of ecosystem succession using the global warming potential of N_2O. For a full explanation of the differences between these calculations of the net CO_2-N_2O effect see the Methods. Units are the net radiative forcing from CO_2 and N_2O (W per m^2 of the Earth's surface) for N-fixing trees relative to non-fixing trees per ha of forest.

Supplementary Table 1: Modeled global net CO_2-N_2O effect of forests and of N-fixing trees relative to non-fixing trees under current N deposition rates from Dentener et al. 2006[1] and Vet et al. 2014[2]

Global forest composition	Global net CO_2-N_2O effect of forests (Pg C yr^{-1})	Global net CO_2-N_2O effect of N-fixing trees (Pg C yr^{-1})	Global net CO_2-N_2O effect of forests (Pg C yr^{-1})	Global net CO_2-N_2O effect of N-fixing trees (Pg C yr^{-1})
N deposition rate reference	Dentener et al. 2006	Dentener et al. 2006	Vet et al. 2014	Vet et al. 2014
Obligate N-fixer and non-fixer	-3.13	+0.50	-3.24	-0.25
Facultative N-fixer and non-fixer	-3.63	+0.002	-3.34	-0.35
Incomplete regulator and non-fixer	-3.54	+0.09	-3.64	-0.65
Non-fixer	-3.63	NA	-2.99	NA

Scenarios displayed are: All N-fixing trees are obligate, all N-fixing trees are facultative, and all N-fixing trees are incomplete regulators. Units are C radiative equivalents, which balance the greenhouse effects of CO_2 and N_2O using the global warming potential of N_2O. Results are comparable to those of Pan et al. 2011[3] which finds that the current annual forest CO_2 sink is 2.4 Pg C yr^{-1}.

Supplementary Table 2: Descriptions, units, values and references of parameters

Parameter	Description	Unit	Value	Reference
ω_0	Nutrient use efficiency of non-fixers	kg C kg N^{-1}	217	Calculated from Batterman et al. 2013[4].
ω_F	Nutrient use efficiency of N-fixers	kg C kg N^{-1}	108	Calculated from Batterman et al. 2013[4] and Adams et al. 2016[5].
v_0	Uptake rate of non-fixers	ha kg C^{-1} y^{-1}	0.2	From Menge et al. 2009[6].
v_F	Uptake rate of N-fixers	ha kg C^{-1} y^{-1}	0.2	From Menge et al. 2009[6].
μ_0	Turnover rate of non-fixers	y^{-1}	0.043	Calculated from Batterman et al. 2013[4].
μ_F	Turnover rate of N-fixers	y^{-1}	0.043	Calculated from Batterman et al. 2013[4].
m	Mineralization rate	y^{-1}	0.2	From Batterman et al. 2013[4].
φ	Unavailable soil N loss rate	y^{-1}	0.001	From Menge et al. 2009[6].
I	Abiotic N input flux	kg N ha^{-1} y^{-1}	See Supp. Table 3.	See Supp. Table 3.
k	Available soil N loss rate (exp. N_2O)	y^{-1}	3.47	Calculated from Menge et al. 2009[6].
β_0	Maximum growth rate of non-fixers	y^{-1}	5.1 (tropical)<br>4.1 (temperate)<br>4.1 (boreal)	Calculated from Batterman et al. 2013[4], Menge and Chazdon 2016[7], Liao et al. 2016[8] and Pregitzer et al. 2004[9].
β_F	Maximum growth rate of N-fixers	y^{-1}	5.6 (tropical)<br>3.7 (temperate)<br>3.7 (boreal)	Calculated from Batterman et al. 2013[4], Menge and Chazdon 2016[7], Liao et al. 2016[8] and Pregitzer et al. 2004[9].
γ_0	Competition coefficient on non-fixers	ha kg C^{-1}	0.00094 (tropical)<br>0.00066 (temperate)<br>0.0013 (boreal)	Calculated from Batterman et al. 2013[4], Menge and Chazdon 2016[7] and Pregitzer et al. 2004[9].
γ_F	Competition coefficient on N-fixers	ha kg C^{-1}	0.0024 (tropical)<br>0.0016 (temperate)<br>0.0031 (boreal)	Calculated from Batterman et al. 2013[4], Menge and Chazdon 2016[7] and Pregitzer et al. 2004[9].
η	N_2O loss rate	y^{-1}	1.53 (tropical)<br>0.04 (temperate)	Calculated from De Klein et al. 2006[10] and Keller and

			0.04 (boreal)	Reiners 1994[11].
ψ	Atmospheric N_2O removal rate	y^{-1}	0.001	From Myhre et al. 2013[12].
F_{max}	Maximum N fixation rate	kg N kg C^{-1} y^{-1}	0.01	From Menge et al. 2009[6].

Supplementary Table 3: N deposition rates

Parameter	Unit	Value	Reference
Low N Deposition Rate	kg N ha^{-1} y^{-1}	0.5	From Galloway et al. 2008[13].
Intermediate N Deposition Rate	kg N ha^{-1} y^{-1}	4.68 (tropical)<br>6.84 (temperate)<br>1.63 (boreal)	From Dentener et al. 2006[1].
		4.06 (tropical)<br>4.43 (temperate)<br>6.04 (boreal)	From Vet et al. 2014[2].
High N Deposition Rate	kg N ha^{-1} y^{-1}	7.20 (tropical)<br>9.18 (temperate)<br>2.15 (boreal)	From Dentener et al. 2006[1].

Supplementary Table 4: Initial pool sizes

Soil N Pool Size	B_F (kg C ha^{-1})	B_0 (kg C ha^{-1})	A (kg N ha^{-1})	D (kg N ha^{-1})	E (kg N_2O-N ha^{-1})
Low	1	1	0	0.5 D^*	0
Intermediate	1	1	0.5 A^*	0.75 D^*	0
High	1	1	A^*	D^*	0

A^* and D^* are the equilibria of A and D respectively (see Supplementary Note 1).

Supplementary Table 5: Descriptions, units, values and references of parameters for the calculation of radiative forcing

Parameter	Description	Unit	Value	Reference
a_0	Fraction of emission that remains in the atmosphere	unitless	0.217	From Alvarez et al. 2012[14].
a_1	Fraction of emission associated with $\tau_{CO_2,1}$	unitless	0.259	From Alvarez et al. 2012[14].
a_2	Fraction of emission associated with $\tau_{CO_2,2}$	unitless	0.338	From Alvarez et al. 2012[14].
a_3	Fraction of emission associated with $\tau_{CO_2,3}$	unitless	0.186	From Alvarez et al. 2012[14].
$\tau_{CO_2,1}$	Time scale 1 of CO_2	yr	172.9	From Alvarez et al. 2012[14].
$\tau_{CO_2,2}$	Time scale 2 of CO_2	yr	18.51	From Alvarez et al. 2012[14].
$\tau_{CO_2,3}$	Time scale 3 of CO_2	yr	1.186	From Alvarez et al. 2012[14].
RE_{N_2O}	Radiative forcing of N_2O	W m^{-2} kg^{-1}	3.85 10^{-13}	Calculated in Equation (13).
RE_{CO_2}	Radiative forcing of CO_2	W m^{-2} kg^{-1}	1.76 10^{-15}	Calculated in Equation (13).
$RE_{N_2O,ppbv}$	Radiative forcing of N_2O	W m^{-2} $ppbv^{-1}$	3.00 10^{-3}	From Myhre et al. 2013[12].
$RE_{CO_2,ppbv}$	Radiative forcing of CO_2	W m^{-2} $ppbv^{-1}$	1.37 10^{-5}	From Myhre et al. 2013[12].
M_A	Mean molecular mass of air	g mol^{-1}	28.97	From Myhre et al. 2013[12].
M_{N_2O}	Molecular mass of N_2O	g mol^{-1}	44.013	
M_{CO_2}	Molecular mass of CO_2	g mol^{-1}	44.01	
T_M	Total mass of	kg	5.14×10^{18}	From Myhre et al. 2013[12].

	atmosphere			

Supplementary Table 6: Modeled global net CO_2-N_2O effect of forests and of N-fixing trees relative to non-fixing trees under future N deposition rates (2030 for the SRES A2 scenario)

	Global Net CO_2-N_2O Effect of Forests (W m^{-2})	Global Net CO_2-N_2O Effect of N-Fixing Trees (W m^{-2})
Obligate N-fixer and non-fixer	-0.0068	+0.0015
Facultative N-fixer and non-fixer	-0.0082	+0.001
Incomplete regulator and non-fixer	-0.0081	+0.001
Non-fixer	-0.0083	NA

Scenarios displayed are: All N-fixing trees are obligate, all N-fixing trees are facultative, and all N-fixing trees are incomplete regulators. CO_2 and N_2O effects are given as radiative forcings.

Supplementary Note 1: Equilibria analysis

The following are the equilibria of the five pools of the model under the assumption that the non-fixer is not N-limited (i.e. $g_0 = \frac{\beta_0}{1+\gamma_0(B_{\mathrm{F}}+B_0)}$) and that $B_{\mathrm{F}}^* = 0$ and $B_0^* > 0$:

$$B_{\mathrm{F}}^* = 0 \quad (1)$$

$$B_0^* = \frac{\beta_0-\mu_0}{\mu_0\gamma_0} \quad (2)$$

$$D^* = \frac{\beta_0-\mu_0}{\omega_0\gamma_0(m+\phi)} \quad (3)$$

$$A^* = \frac{I\omega_0\gamma_0 m+I\omega_0\gamma_0\phi-\phi\beta_0+\phi\mu_0}{\omega_0\gamma_0(k+\eta)(m+\phi)} \quad (4)$$

$$E^* = \frac{\eta A^*}{\psi} \quad (5)$$

See the Methods and Supplementary Table 2 for descriptions of the parameters.

Analysis of the eigenvalues of the Jacobian matrix of a differential equation model is the standard technique in theoretical ecology for assessing the local asymptotic stability of equilibria[15]. The Jacobian matrix of the model evaluated at this equilibrium is:

$$J = \begin{bmatrix} \frac{(\beta_{\mathrm{F}}-\mu_{\mathrm{F}})\gamma_0\mu_0-(\beta_0-\mu_0)\gamma_{\mathrm{F}}\mu_{\mathrm{F}}}{(\beta_0-\mu_0)\gamma_{\mathrm{F}}+\gamma_0\mu_0} & 0 & 0 & 0 & 0 \\ \frac{(\mu_0-\beta_0)\mu_0}{\beta_0} & \frac{(\mu_0-\beta_0)\mu_0}{\beta_0} & 0 & 0 & 0 \\ \frac{\mu_{\mathrm{F}}}{\omega_{\mathrm{F}}} & \frac{\mu_0}{\omega_0} & -m-\phi & 0 & 0 \\ \frac{-\omega_{\mathrm{F}}(\gamma_0-\gamma_{\mathrm{F}})\mu_0^3+\beta_0\omega_{\mathrm{F}}(\gamma_0-2\gamma_{\mathrm{F}})\mu_0^2+\left((\gamma_{\mathrm{F}}\beta_0+F\omega_0(\gamma_0-\gamma_{\mathrm{F}}))\omega_{\mathrm{F}}-\beta_{\mathrm{F}}\gamma_0\omega_0\right)\beta_0\mu_0+F\beta_0^2\gamma_{\mathrm{F}}\omega_0\omega_{\mathrm{F}}}{\omega_0\omega_{\mathrm{F}}\beta_0((\gamma_0-\gamma_{\mathrm{F}})\mu_0+\gamma_{\mathrm{F}}\beta_0)} & \frac{-\mu_0^2}{\beta_0\omega_0} & m & -k-\eta & 0 \\ 0 & 0 & 0 & \eta & -\psi \end{bmatrix} \quad (6)$$

The eigenvalues of the Jacobian matrix are $\lambda_1 = \frac{(\beta_{\mathrm{F}}-\mu_{\mathrm{F}})\gamma_0\mu_0-(\beta_0-\mu_0)\gamma_{\mathrm{F}}\mu_{\mathrm{F}}}{(\beta_0-\mu_0)\gamma_{\mathrm{F}}+\gamma_0\mu_0}$, $\lambda_2 = \frac{(\mu_0-\beta_0)\mu_0}{\beta_0}$, $\lambda_3 = -m-\phi$, $\lambda_4 = -k-\eta$ and $\lambda_5 = -\psi$. The equilibrium is stable if $\frac{\beta_{\mathrm{F}}-\mu_{\mathrm{F}}}{\gamma_{\mathrm{F}}\mu_{\mathrm{F}}} < \frac{\beta_0-\mu_0}{\gamma_0\mu_0}$ and $\mu_0 < \beta_0$. For our parameter values, the equilibrium is stable. A second equilibrium exists for which $B_{\mathrm{F}}^* > 0$

and $B_0^* = 0$. The analysis of this equilibrium was omitted because, for our parameter values, this equilibrium is unstable.

The following are the equilibria of the five pools of the model under the assumption that the non-fixer is N-limited (i.e. $g_0 = \omega_0 \nu_0 A$) and that $B_F^* = 0$ and $B_0^* > 0$:

$$B_{\mathrm{F}}^* = 0 \tag{7}$$

$$B_0^* = \frac{I\omega_0 v_0(m+\phi) - \mu_0(k+\eta)(m+\phi)}{\phi \mu_0 v_0} \tag{8}$$

$$D^* = \frac{I\omega_0 v_0 - \mu_0(k+\eta)}{\phi \omega_0 v_0} \tag{9}$$

$$A^* = \frac{\mu_0}{\omega_0 v_0} \tag{10}$$

$$E^* = \frac{\eta A^*}{\psi} \tag{11}$$

The Jacobian matrix of the model evaluated at this equilibrium is:

$$J = \begin{bmatrix} \frac{v_0 \omega_{\mathrm{F}} F - \mu_{\mathrm{F}} v_0 \omega_0 + \mu_0 v_{\mathrm{F}} \omega_{\mathrm{F}}}{\omega_0 v_0} & 0 & 0 & 0 & 0 \\ 0 & 0 & 0 & -\frac{\omega_0(m+\phi)\left((\eta+k)\mu_0 - I\omega_0 v_0\right)}{\phi \mu_0} & 0 \\ \frac{\mu_{\mathrm{F}}}{\omega_{\mathrm{F}}} & \frac{\mu_0}{\omega_0} & -m-\phi & 0 & 0 \\ -\frac{\mu_0 v_{\mathrm{F}}}{\omega_0 v_0} & -\frac{\mu_0}{\omega_0} & m & -\frac{\mu_0 m(\eta+k) - I\omega_0 v_0(m+\phi)}{\phi \mu_0} & 0 \\ 0 & 0 & 0 & \eta & -\psi \end{bmatrix} \tag{12}$$

The eigenvalues are omitted due to their complexity. For our parameter values, the equilibrium is stable. A second equilibrium exists for which $B_0^* = 0$ and $B_F^* > 0$. The analysis of this equilibrium was omitted because, for our parameter values, this equilibrium is unstable.

Supplementary References

1. Dentener, F. *et al.* Nitrogen and sulfur deposition on regional and global scales: A multimodel evaluation. *Global Biogeochem. Cycles* **20,** 1–21 (2006).
2. Vet, R. *et al.* A global assessment of precipitation chemistry and deposition of sulfur, nitrogen, sea salt, base cations, organic acids, acidity and pH, and phosphorus. *Atmos. Environ.* **93**, 3–100 (2014).
3. Pan, Y. *et al.* A Large and Persistent Carbon Sink in the World's Forests. *Science* **333,** 988–993 (2011).
4. Batterman, S. A. *et al.* Key role of symbiotic dinitrogen fixation in tropical forest secondary succession. *Nature* **502,** 224–227 (2013).
5. Adams, M. A., Turnbull, T. L., Sprent, J. I. & Buchmann, N. Legumes are different: Leaf nitrogen, photosynthesis, and water use efficiency. *Proc. Natl. Acad. Sci.* **113,** 4098–4103 (2016).
6. Menge, D. N. L., Levin, S. A. & Hedin, L. O. Facultative versus Obligate Nitrogen Fixation Strategies and Their Ecosystem Consequences. *Am. Nat.* **174,** 465–477 (2009).
7. Menge, D. N. L. & Chazdon, R. L. Higher survival drives the success of nitrogen-fixing trees through succession in Costa Rican rainforests. *New Phytol.* **209,** 965–977 (2016).
8. Liao, W. & Menge, D. N. L. Demography of symbiotic nitrogen-fixing trees explains their rarity and successional decline in temperate forests in the United States. *PLoS One* https://doi.org/10.1371/journal.pone.0164522 (2016).
9. Pregitzer, K. S. & Euskirchen, E. S. Carbon cycling and storage in world forests: Biome patterns related to forest age. *Glob. Chang. Biol.* **10,** 2052–2077 (2004).
10. De Klein, C. *et al.* in *IPCC Guidelines for National Greenhouse Gas Inventories* (eds

Eggleston, H. S. *et al.*) Vol. 4 11.1-11.54 (IPCC, IGES, 2006).

11. Keller, M. & Reiners, W. A. Soil-atmosphere exchange of nitrous oxide, nitric oxide, and methane under secondary succession of pasture to forest in the Atlantic lowlands of Costa Rica. *Global Biogeochem. Cycles* **8,** 399–409 (1994).

12. Myhre, G. *et al.* in *Climate Change 2013: The Physical Science Basis* (eds Stocker, T. *et al.*) 659-740 (IPCC, Cambridge Univ. Press, 2013).

13. Galloway, J. N. *et al.* Transformation of the Nitrogen Cycle: Recent Trends, Questions, and Potential Solutions. *Science* **320,** 889–892 (2008).

14. Alvarez, R. A., Pacala, S. W., Winebrake, J. J., Chameides, W. L. & Hamburg, S. P. Greater focus needed on methane leakage from natural gas infrastructure. *Proc. Natl. Acad. Sci.* **109,** 6435–6440 (2012).

15. Hastings, A. *Population Biology Concepts and Models* (eds Hastings, A.) (Springer, 1997).
